# Recurrent fever-associated acute liver failure and cranial dysmorphism in children caused by RINT1 gene mutations: a rare case report

**DOI:** 10.3389/fped.2025.1698931

**Published:** 2025-10-13

**Authors:** Yanfei Cui, Fawudan Abudu, Yipaguli Simijiang

**Affiliations:** Department of Critical Care Medicine, Pediatric Research Institute of Xinjiang Uygur Autonomous Region, Children’s Hospital of Xinjiang Uygur Autonomous Region, Xinjiang Hospital of Beijing Children’s Hospital, The Seventh People’s Hospital of Xinjiang Uygur Autonomous Region, Urumqi, Xinjiang, China

**Keywords:** RINT1 gene, acute liver failure, infantile liver failure syndrome type 3, coagulation dysfunction, cranial dysmorphism

## Abstract

Mutations in the RINT1 gene represent a rare genetic cause of recurrent fever-associated acute liver failure (ALF) accompanied by skeletal abnormalities in infants and children. We report the case of a 9-month-old infant presenting with multisystem involvement, primarily characterized by recurrent fever-associated ALF and cranial dysmorphism, due to compound heterozygous mutations in the RINT1 gene. The patient exhibited abnormal liver function tests and coagulation dysfunction following febrile episodes. Over a period of more than one year, the patient initially experienced two episodes of acute liver injury, followed by two episodes of ALF, with progressively worsening clinical manifestations. Whole-exome sequencing (WES) identified compound heterozygous variants in the RINT1 gene (exons 12–14 deletion; intron 11, c.1672-1G > T, p.?), consistent with a diagnosis of infantile liver failure syndrome-3 (ILFS3). Between episodes, liver function failed to return fully to baseline and was accompanied by growth retardation, delayed psychomotor development, cranial dysmorphism, and beak-like deformities of vertebral bodies. This case highlights the critical role of RINT1 mutations in the pathogenesis of recurrent fever-associated ALF and emphasizes the importance of recognizing associated skeletal developmental abnormalities, including cranial dysmorphism. Early genetic diagnosis and prompt antipyretic intervention may mitigate liver injury and improve long-term outcomes. By documenting cranial dysmorphism in this context, we aim to expand the recognized phenotypic spectrum of ILFS3 and improve clinical awareness among pediatricians and geneticists.

## Introduction

Acute liver failure (ALF) in children is an uncommon but life-threatening condition that requires prompt diagnosis and management. The etiologies of pediatric ALF are diverse and include infections, shock, drug or toxin exposure, autoimmune disorders, and inborn errors of metabolism (IEMs). Despite extensive evaluation, the underlying cause remains unidentified in a subset of cases (1). Among the genetic and metabolic causes of recurrent acute liver failure (RALF), notable examples include leucyl-tRNA synthetase deficiency caused by mutations in the LARS gene, also referred to as infantile liver failure syndrome type 1 (ILFS1) (2); infantile liver failure syndrome type 2 (ILFS2), resulting from mutations in the NBAS gene (3–5); RINT1 deficiency (1); and various mitochondrial disorders (6).

In children with certain hereditary liver diseases, RALF is consistently triggered by fever, and each episode may be partially ameliorated by supportive interventions such as antipyretic therapy and hepatoprotective measures. The currently identified pathogenic genes associated with fever-induced RALF include NBAS, SCYL1, and RINT1 (7). Recent studies have demonstrated that RINT1 mutations disrupt Golgi-endoplasmic reticulum retrograde transport and autophagy, resulting in fever-associated ALF accompanied by skeletal dysplasia (1, 8). Since its first report in 2019, RINT1-related ALF remains extremely rare, with only a handful of cases documented worldwide. Notably, cranial dysmorphism has not previously been reported in these patients.

Here, we present the case of an infant with recurrent fever-associated ALF due to RINT1 mutations, who also displayed cranial dysmorphism and vertebral anomalies. By reporting this case, we aim to expand the known clinical spectrum of RINT1-associated disease, emphasize the importance of early genetic testing in infants with recurrent ALF, and highlight the potential benefits of timely antipyretic management in improving prognosis. This case underscores the need for heightened awareness among clinicians regarding the multisystem manifestations of RINT1 mutations, which extend beyond the liver to skeletal and neurodevelopmental systems.

## Case presentation

The patient was the second child of a non-consanguineous couple and was delivered at 38 weeks of gestation via spontaneous vaginal birth. His birth weight was 2.0 kg (<3rd percentile) and length was 45 cm (<3rd percentile), consistent with a diagnosis of small for gestational age (SGA). The Apgar score at birth was not documented. Shortly after birth, he was admitted to the neonatal unit for 8 days due to low birth weight and suspected intrauterine infection. He was exclusively breastfed, but because of weak sucking ability, each feeding required 30–40 min. Complementary foods were introduced at 5 months of age; however, his daily intake of both milk and solid foods remained below that of age-matched peers. At home, he was primarily cared for by his mother and grandmother, who engaged in daily stimulation activities such as playing music, talking, interactive play, and performing physical rehabilitation exercises modeled after professional therapy techniques. When his condition was stable, he attended regular outpatient follow-up visits. During febrile episodes, the caregivers initiated physical cooling measures and promptly sought medical attention. From early infancy, he demonstrated progressive delays in cognitive, motor, language, and overall growth development. Despite routine rehabilitation therapy, improvements were minimal. Developmental milestones were significantly delayed: although he achieved head control at 4 months, by 2 years and 6 months he remained unable to sit independently or walk. His expressive language was very limited, consisting only of occasional words such as “papa” and “mama,” and he primarily relied on gestures for communication.

**Figure 1:**
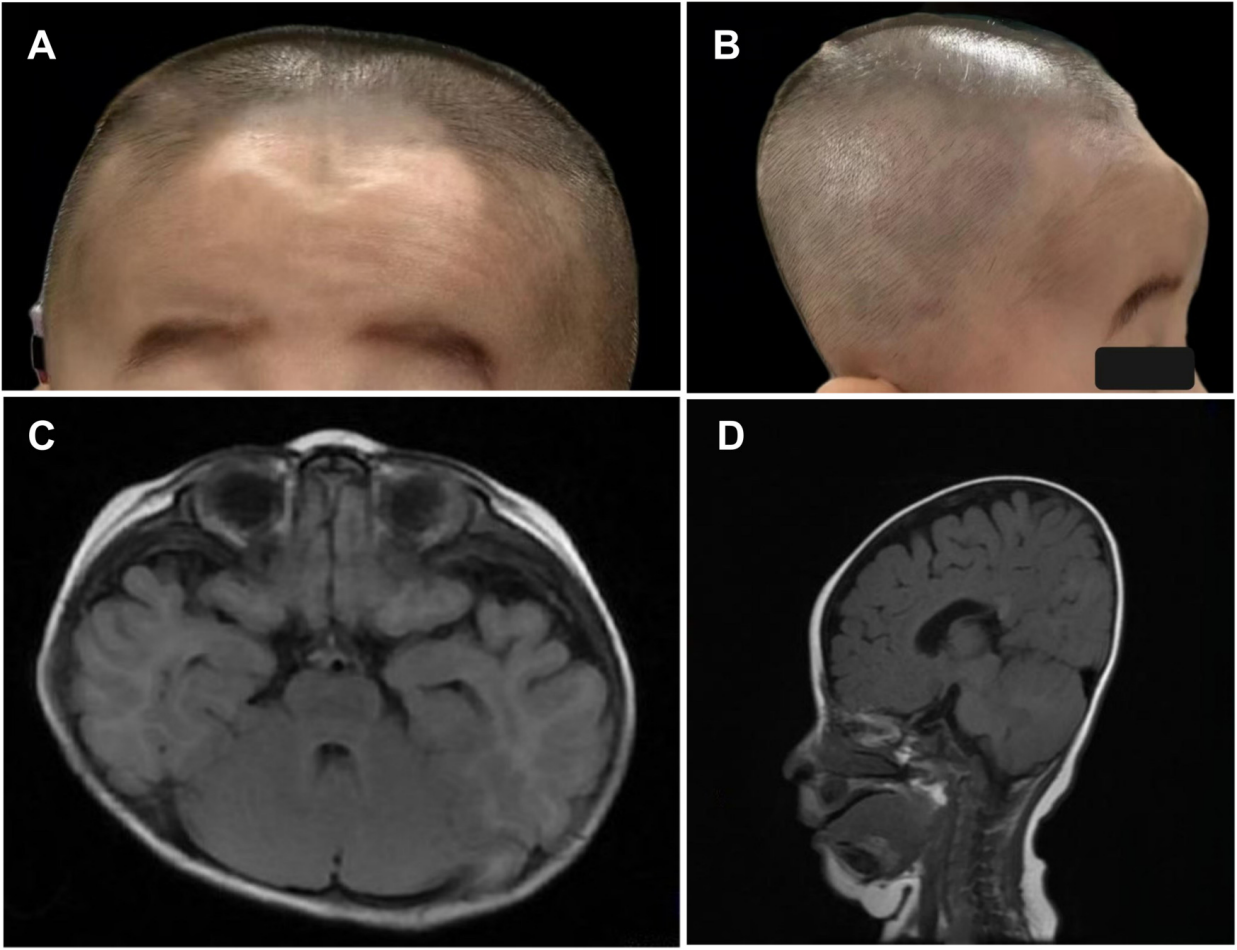
**(A–D)** photographs and cranial magnetic resonance imaging (MRI) of the patient demonstrate skull dysplasia, characterized by a shortened anteroposterior diameter and an elongated left-right diameter of the skull.

**Figure 2:**
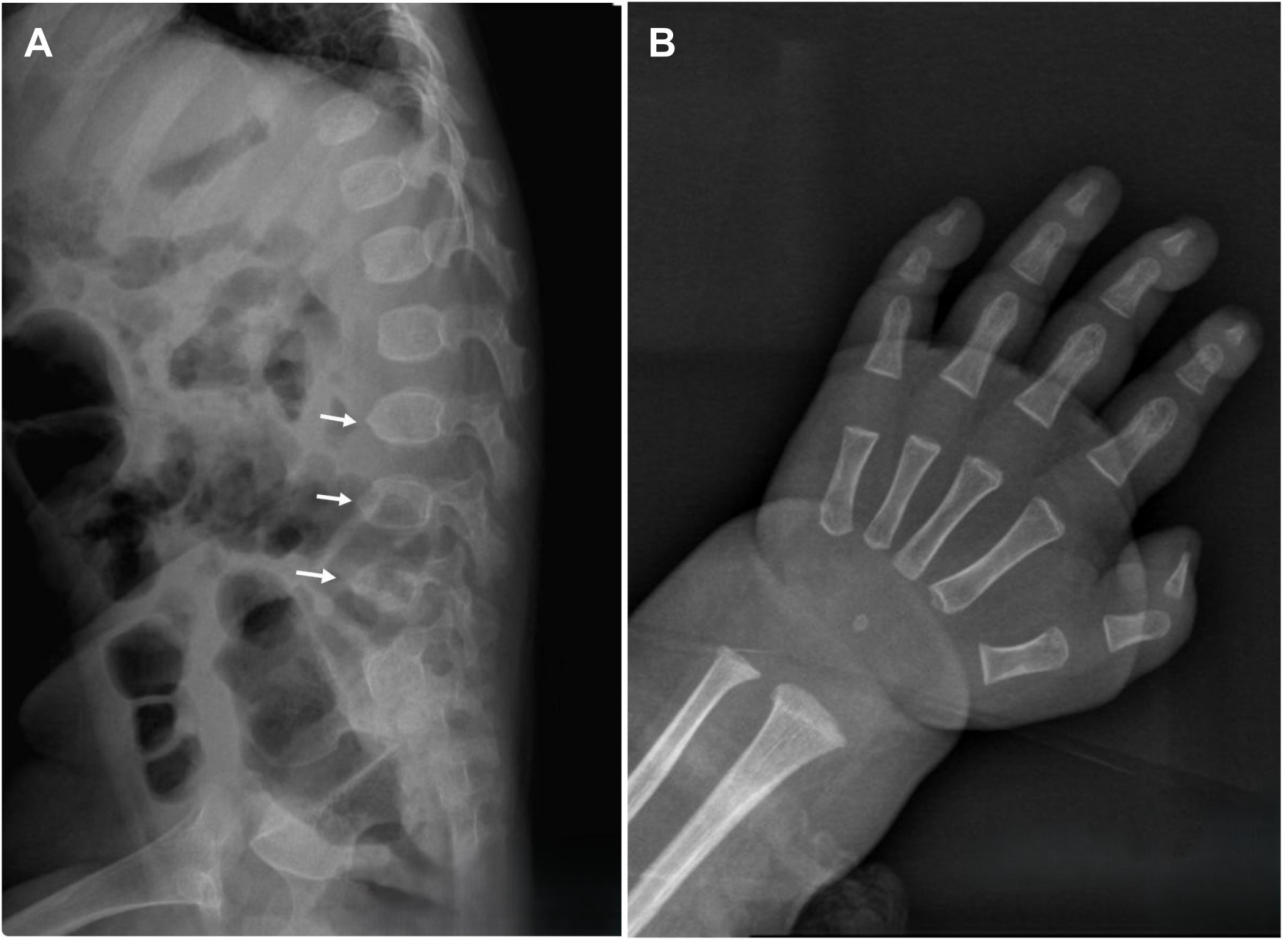
**A** Lumbar spine x-ray reveals localized lumbar kyphosis, with beak-like deformities at the anterior edges of the third, fourth, and fifth lumbar vertebrae (indicated by white arrows). **(B)** Bone age radiograph at 9 months of age shows a delay of approximately 0.5 years.

On physical examination, the child demonstrated short stature, severe emaciation, and distinctive craniofacial features, including a broad forehead and cranial dysmorphism (Figures 1A,B). Cardiopulmonary examination revealed no abnormalities, and hepatosplenomegaly was absent throughout the disease course. Cranial magnetic resonance imaging (MRI) revealed skull dysplasia, characterized by a shortened anteroposterior diameter and an elongated left-right diameter, resembling brachycephaly associated with bilateral coronal synostosis (Figures 1C,D). However, the cranial sutures were intact, ruling out true coronal synostosis. Lumbar spine x-ray demonstrated localized lumbar kyphosis and beak-like deformities at the anterior margins of the third, fourth, and fifth lumbar vertebrae (Figure 2A). Bone age assessment at 9 months of age revealed a delay of approximately 0.5 years (Figure 2B).

**Figure 3:**
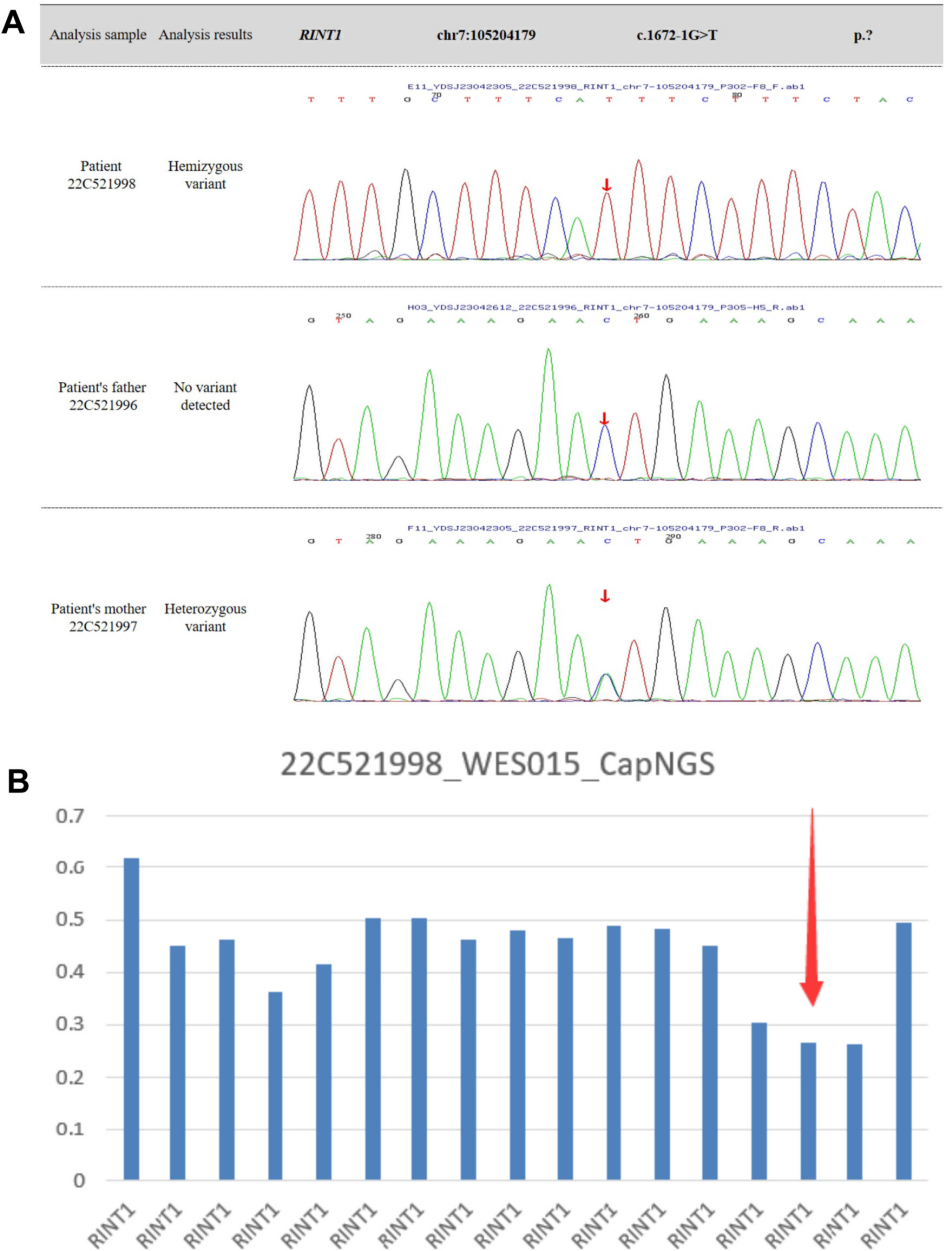
**(A,B)** whole-exome sequencing identifies compound heterozygous variants in the RINT1 gene, including a deletion spanning exons 12-14 and a splice-site variant in intron 11 (c.1672-1G > T, p.?).

The patient experienced fever-associated acute liver injury at 9 and 19 months of age, followed by fever-associated ALF with coagulopathy at 29 and 30 months of age. Whole-exome sequencing (WES) confirmed the diagnosis of infantile liver failure syndrome type 3 (ILFS3), caused by compound heterozygous mutations in the RINT1 gene (Figures 3A,B). Based on the clear clinical history and genetic findings, further metabolic screening was not performed. During each hospitalization, the child presented with post-infectious fever ranging from 38 to 40°C, accompanied by nonspecific symptoms such as lethargy and vomiting. Coagulation abnormalities were refractory to correction with vitamin K. Symptomatic management, including antipyretic therapy, hepatoprotective agents, antiviral and anti-infective treatment, and fresh frozen plasma transfusion, resulted in only partial improvement of liver enzyme levels. However, liver function never fully normalized between episodes, and both hepatic and coagulation dysfunction progressively deteriorated with each recurrence. During the fourth episode, the child developed severe liver failure complicated by hepatorenal syndrome, leading to edema and oliguria, and ultimately died during continuous venovenous hemodiafiltration (CVVHDF).

Notably, the patient had a deceased elder sister who exhibited a similar clinical course. She was the first child of the same couple, born at 39 + 4 weeks of gestation via spontaneous vaginal delivery, with a birth weight of 2.2 kg (<3rd percentile) and a birth length of 47 cm (<10th percentile). She experienced global developmental delay and was admitted to the hospital at 19 months of age with fulminant liver failure, hepatic encephalopathy, and hyperammonemia. She died one day after admission. Her medical history also included episodes of abnormal liver function following fever, although detailed records were unavailable. Genetic testing was not performed due to the rapid progression of her disease and the parents’ decision to decline further evaluation. Detailed clinical and laboratory information for both children is summarized in Table 1.

**Table 1:** Clinical features of the patients.

Clinical features	Patient (male)				Deceased elder sister
	9 months of age	19 months of age	29 months of age	30 months of age	19 months of age
Weight	7.1 kg (<3th percentile)	7.8 kg (<3th percentile)	8.5 kg (<3th percentile)	8.5 kg (<3th percentile)	8.0 kg (<3th percentile)
Height	68 cm (3th percentile)	80 cm (<25th percentile)	82 cm (<3th percentile)	82 cm (<3th percentile)	75 cm (<3th percentile)
Head circumference	–	43 cm (<3th percentile)	–	–	–
Suspected triggers of episodes	Fever or infection	Fever	Fever	Fever	Fever and illness
Glucose	–	4.78 mmol/L(ref:3.9–6.1 mmol/L)	3.72 mmol/L(ref:3.9–6.1 mmol/L)	2.76 mmol/L(ref:3.9–6.1 mmol/L)	2.36 mmol/L(ref:3.9–6.1 mmol/L)
Total bilirubin	11.33 umol/L(ref:0–21 umol/L)	23.08 umol/L(ref:0–21 umol/L)	70.9 umol/L(ref:0–21 umol/L)	91.08 umol/L(ref:0–21 umol/L)	68.5 umol/L(ref:3.4–28 umol/L)
Direct bilirubin	7.16 umol/L(ref:0–6.8 umol/L)	14 umol/L(ref:0–6.8 umol/L)	43.3 umol/L(ref:0–6.8 umol/L)	61.75 umol/L(ref:0–6.8 umol/L)	45.5 umol/L(ref:0–10 umol/L)
Prothrombin time	16s(ref:11.0–13.7 s)	15.6s(ref:11.0–13.7 s)	60.5s(ref:12.1–14.5 s)	98.9s(ref:11.0–13.7 s)	80.3s(ref:11.0–14.3 s)
INR	1.4(ref:0.8–1.2)	1.4(ref:0.8–1.2)	7.3(ref:0.92–1.14)	8.6(ref:0.8–1.2)	7(ref:0.8–1.2)
Highest AST/ALT (U/L) during ALF	2,461/1,902(ref:8–71/21–80)	4,161/7,156(ref:8–42/22–59)	4,867/1,426(ref:7–30/14–44)	4,438/17,367(ref:7–30/14–44)	12,688/21,035(ref:8–42/22–59)
Lowest AST/ALT (U/L) between episodes	286/47(ref:8–71/21–80)	273/74(ref:8–42/22–59)	84/39(ref:7–30/14–44)	825/178(ref:7–30/14–44)	–

## Discussion

Recurrent fever-associated ALF in children is an extremely rare and potentially life-threatening condition, predominantly triggered by post-infectious fever. It is characterized by repeated episodes of acute liver injury, during which liver enzyme levels may partially or completely return to normal between episodes. This cyclical pattern distinguishes genetic or hereditary forms of ALF from other more common acute liver insults. To date, several gene mutations have been identified as causative factors for recurrent fever-associated ALF, including NBAS, SCYL1, and RINT1 (1). The RINT1 gene, located on chromosome 7q22.3, has been associated with additional disorders beyond ALF, including early-onset breast cancer, hereditary spastic paraplegia, and cancers within the Lynch syndrome spectrum (9, 10). These findings underscore the diverse biological roles of RINT1 in cellular processes such as vesicular transport, DNA damage response, and genomic stability.

In 2019, Cousin et al. (1) first reported three pediatric cases of recurrent fever-associated ALF accompanied by skeletal abnormalities caused by RINT1 mutations, designating this condition as infantile liver failure syndrome type 3 (ILFS3). Their study suggested that missense mutations, in-frame deletions, and other variants affecting conserved residues in the RINT1 protein lead to a significant decrease in protein levels, impairing its normal cellular function. Functional studies in fibroblasts have demonstrated that RINT1 interacts with NBAS and UV radiation resistance-associated gene protein (UVRAG) to mediate retrograde vesicular transport from the Golgi apparatus to the endoplasmic reticulum (11). In cases involving compound heterozygous mutations, RINT1 protein levels are drastically reduced, and the remaining protein exhibits thermal instability. During febrile episodes, this instability is further exacerbated, resulting in fragmentation of the Golgi apparatus, impaired vesicular transport, and disruption of the anterograde trafficking of type VII collagen. These cellular dysfunctions can lead to hepatocyte injury, acute liver failure, and skeletal abnormalities resembling lysosomal storage disorders (1, 12–14). Despite these insights, the precise molecular and cellular mechanisms responsible for the diverse phenotypes observed in ILFS3 remain incompletely understood, and further experimental studies are required to fully elucidate the pathophysiology of this condition.

The clinical manifestations observed in the present patient, including markedly elevated alanine aminotransferase (ALT) and aspartate aminotransferase (AST), severe coagulopathy, hyperbilirubinemia, hypoglycemia, hyperammonemia, and episodes of hepatic encephalopathy, are consistent with the spectrum reported by Cousin et al. (1). Skeletal abnormalities in ILFS3 differ notably from those observed in NBAS-related disease, which often presents with features of atypical osteogenesis imperfecta such as bone fragility, multiple fractures, and abnormally slender long bones. By contrast, skeletal changes associated with RINT1 mutations predominantly involve vertebral dysplasia, acetabular dysplasia, and irregular femoral head epiphyses, suggesting distinct underlying pathobiological mechanisms (1). The patient described in this report exhibited lumbar kyphosis and beak-like deformities of the third to fifth lumbar vertebrae, consistent with previous cases. Importantly, this patient also presented with cranial dysmorphism and delayed bone age, features that have not been previously documented in the literature. Cranial MRI excluded the presence of coronal synostosis, and positional effects were ruled out, indicating that these findings are likely directly attributable to RINT1-related skeletal pathology. These observations expand the recognized phenotypic spectrum of ILFS3 and highlight the potential for previously unrecognized craniofacial involvement in affected children.

Due to the absence of specific biomarkers, the diagnosis of ILFS3 currently relies on the recognition of characteristic clinical features and confirmation through genetic testing. Children presenting with severe liver dysfunction or ALF triggered by febrile episodes, particularly when accompanied by skeletal abnormalities, should undergo prompt genetic evaluation after exclusion of common infectious, autoimmune, and toxic etiologies. Currently, there is no disease-specific treatment for ILFS3, and formal clinical management guidelines are lacking. Supportive therapy remains the mainstay of care. Based on the proposed pathophysiological mechanisms, aggressive antipyretic therapy and glucose supplementation during acute episodes are critically important for stabilizing liver function (15). Additionally, the use of hepatoprotective and antioxidant agents may aid recovery, while fever prevention during inter-episode periods is essential for long-term disease management. In the case presented here, despite the implementation of appropriate supportive care during each hospitalization, the child's condition progressively worsened, illustrating the severe and refractory nature of this disease. Liver transplantation was not pursued due to financial constraints, and its efficacy in ILFS3 remains undetermined. Ongoing advancements in medical science and gene-targeted therapies may eventually provide more effective treatment options for affected patients.

Since the first description of RINT1-related ILFS3 in 2019 (1), only a limited number of cases have been reported worldwide, and the long-term prognosis remains uncertain. Some previously reported patients demonstrate partial improvement with age (1), suggesting that the disease course may be influenced by developmental and environmental factors. In contrast, the two siblings described in this study experienced progressive deterioration and ultimately succumbed to liver failure. This variability in disease severity may reflect differences in the underlying RINT1 variants, although additional studies are required to confirm genotype-phenotype correlations. Whole-exome sequencing of the younger sibling revealed compound heterozygous RINT1 variants, confirming the diagnosis of ILFS3. Unfortunately, functional studies to verify the effect of these variants on protein structure and stability were not performed due to resource limitations. The elder sibling was highly suspected to have suffered from the same genetic condition, but genetic testing was not conducted. Consequently, the mother underwent a subsequent pregnancy without prior genetic counseling, leading to the birth of a second affected child. This tragic sequence of events underscores the critical importance of early genetic testing and comprehensive genetic counseling for families with a history of unexplained recurrent ALF. Identification of RINT1 mutations could enable families to make informed reproductive decisions, provide prenatal diagnostic options, and potentially prevent recurrence of severe disease in future offspring.

## Conclusion

In most previously reported cases of ILFS3, skeletal abnormalities were primarily limited to acetabular dysplasia and beak-like vertebral changes. In this report, cranial dysmorphism and delayed bone age are described for the first time as potential phenotypic features of RINT1-related disease. The underlying mechanisms remain unclear, but they may share similarities with other RINT1-related skeletal abnormalities. This case expands the clinical spectrum of ILFS3 and highlights the importance of considering genetic causes in children with recurrent fever-associated liver failure and skeletal anomalies. Furthermore, the familial occurrence in this report illustrates the critical role of genetic testing and counseling in preventing recurrence and informing management decisions. Future studies are needed to clarify the molecular mechanisms of cranial and skeletal involvement, improve therapeutic strategies, and establish guidelines for early diagnosis and intervention in RINT1-associated ILFS3.

## Data Availability

The original contributions presented in the study are included in the article/Supplementary Material, further inquiries can be directed to the corresponding author.
